# Examining the influence of wealth status on prehypertension risk in women aged 30–49: evidence from the 2018 Benin demographic and health survey

**DOI:** 10.1186/s13104-023-06676-6

**Published:** 2024-01-02

**Authors:** Castro Ayebeng, Joshua Okyere, Samuel Salu, Kwamena Sekyi Dickson

**Affiliations:** 1 Department of Population and Health, University of Cape Coast, Cape Coast, Ghana; 2 School of Nursing & Midwifery, College of Health Sciences, Kwame Nkrumah University of Science and Technology, Kumasi, Ghana; 3 Department of Epidemiology and Biostatistics, University of Health and Allied Sciences, Ho, Ghana

**Keywords:** Prehypertension, Wealth status, Benin, Demographic and health survey, Non-communicable diseases

## Abstract

**Background:**

There is an interest in the extent to which the wealth status of women predicts their risk of being pre-hypertensive. This understanding is lacking in the current body of empirical literature, particularly within the context of Benin. Thus, indicating a knowledge gap that must be filled. To this end, the present study aimed to assess the association between wealth status and the risk of prehypertension among women aged 30–49 years in Benin.

**Methods:**

This study used a secondary data from the recent (2018) Demographic and Health Survey of Benin. Bivariate and multivariate logistic regression models were computed to examine the association between wealth index and the risks of prehypertension using Stata version 14. Findings were presented in adjusted odds ratio at 95% confidence level.

**Results:**

Women in the richest wealth index were significantly more likely to have prehypertension than those in the poorest wealth index [AOR = 1.4; 95%CI: 1.26–2.26]. Women aged 45–49 years were more likely to have prehypertension [AOR = 1.5; 95%CI: 1.15–1.98] compared to younger women. Women who used unclean cooking fuel were less likely to have prehypertension compared to those using clean cooking fuel [AOR = 0.6; 95%CI: 0.37,0.87].

**Conclusion:**

The study concludes that wealth status is a significant predictor of prehypertension among women in Benin. Therefore, the Ministry of Health in Benin should prioritize health education and prehypertension awareness campaigns specifically targeting women in affluent communities and households. These campaigns should focus on promoting healthy dietary choices and encouraging physical activity to mitigate the elevated risk associated with wealth status. Recognizing the influence of age on prehypertension risk, it is imperative for older-middle aged women to be targeted as primary beneficiaries of health education programs and prehypertension screening programs.

## Background

The ageing population of the world coupled with an increase in sedentary lifestyles and poor dietary habits has culminated in exacerbating the incidence of cardiovascular diseases [1, 2]. High blood pressure is one of the common related cardiovascular health problems affecting millions of people worldwide. According to the World Health Organization (WHO) [3], an adult has high blood pressure also known as hypertension when the readings show a systolic and diastolic being 140 mm Hg over and 90 mm Hg over, respectively. Below this range, an adult is considered to have a normal blood pressure (120/80mmHg). However, studies have shown that before becoming hypertensive, individuals experience what is known as pre-hypertension [4–6]. That is, a state where a person is neither hypertensive nor has normal blood pressure. In terms of its measurement, pre-hypertension is described as having a systolic blood pressure between 120 mmHg and 139 mmHg, and a diastolic blood pressure of 80 mmHg [6].

Reports from the WHO [3] indicate that there were approximately 1.28 billion adults living with hypertension in the world; of this number, nearly half (46%) were unaware of their status. This is why hypertension has infamously been tagged as the silent killer. It is estimated that the prevalence of hypertension will increase by 60% to 1.56 billion cases by the end of 2025 [7]. In sub-Saharan Africa, more than 50% of adults are hypertensive [8]. A systematic review has reported that in Africa, the prevalence of prehypertension ranges between 32.9 and 56.8% among adults while ranging between 2.5 and 34% in children and adolescents [9]. While the prevalence of prehypertension in Benin remains unclear, a study has shown that about 32.9% of the adult population is hypertensive [10]. Thus, making prehypertension and hypertension a serious public health concern in the Republic of Benin.

Exiting empirical studies conducted in Indonesia [5], Algeria [11], Vietnam [12], Cameroon [13], and South Asia [14] have identified several factors to be associated with the risk of prehypertension and hypertension. For instance, Moussouni et al.’s [11] study found ageing, obesity, and abdominal obesity to be the main risk factors of prehypertension and hypertension. Similarly, Kamdem et al. [13] reports that obesity, physical inactivity, and hyperglycemia exacerbated the risk of prehypertension and hypertension among adolescents in Cameroon. Vo et al. [12] also identified alcohol consumption as a key risk factor for prehypertension and hypertension in Vietnam.

Beyond these established risk factors, there is an interest in the extent to which the wealth status of women predicts their risk of being pre-hypertensive. The extant body of literature has inconsistent findings regarding this subject. For instance, while evidence from Algeria [11] shows no significant association, a previous study conducted in South Asia [14] found a positive significant association between wealth status and prehypertension risk. There is also evidence showing that prehypertension risk declines with increasing wealth status [15]. These inconsistencies suggest that there is a need for more research to fully understand the relationship between women’s wealth status and their risk of prehypertension. This understanding is lacking particularly within the context of Benin. Thus, indicating a knowledge gap that must be filled. To this end, the present study aimed to assess the association between wealth status and the risk of prehypertension among women aged 30–49 years in Benin. Knowing how socioeconomic status influences hypertension risk would allow healthcare professionals and policymakers to develop targeted interventions. These interventions can be tailored to address the specific needs of vulnerable populations while ensuring cost-effectiveness in resource allocation in the management of these conditions.

## Hypothesis

We test the hypothesis that wealth status has a significantly positive association with the risk of prehypertension among women aged 30–49 years.

## Methods

This study employed a secondary analysis of nationally representative data from the 2018 Benin Demographic and Health Survey (BDHS). The DHS employed a cross-sectional design to collect information from women, men, and children [16]. The 2017–2018 Benin Demographic and Health Survey (DHS) employed a two-stage cluster sampling approach, with Cotonou, urban areas outside of Cotonou, and rural areas as strata. A total of 555 enumeration areas (EAs) were chosen using the 2013 Benin census frame as a reference, and within each EA, 26 households were randomly selected. Specifically, 52 EAs were designated in the city of Cotonou. Additional details of the sampling technique can be found in previously published sources [16, 17]. Pretested and structured questionnaires were employed to gather data on various health and demographic variables including measures of blood pressure levels. In our study, we used a weighted sample of 2,787 women aged (30–49 years) who had complete data for all the variables of interest in our analysis. We relied on the Strengthening the Reporting of Observational Studies in Epidemiology (STROBE) guidelines in preparing this paper. The dataset used in our study is freely available for download at https://dhsprogram.com/data/dataset/Benin_Standard-DHS_2017.cfm?flag=1.

### Study variables and measurement

#### Outcome variable

This study examined data from 2,787 participants, with 291 individuals excluded due to hypertension (defined as SBP ≥ 140 mmHg, DBP ≥ 90 mmHg, or use of antihypertensive medications) identified during the initial survey. Participants’ systolic and diastolic blood pressure were measured three times while sitting, using a standard digital blood pressure monitor on their right arm, over loose clothing. The mean of these three measurements was utilized for the analysis. Prehypertension was defined as having a systolic blood pressure (SBP) ranging from 120 mmHg to 139 mmHg and/or a diastolic blood pressure (DBP) ranging from 80 mmHg to 89 mmHg, as described by Tamrakar et al. [18].

#### Explanatory variables

The main explanatory variable of this study is wealth index which is categorized into five groups: poorest = 0, poorer = 1, middle = 2, richer = 3, and richest = 4. The wealth index serves as a composite measure of a household’s overall living standard. The data for the wealth index is derived from the Household Questionnaire [19]. This questionnaire includes questions concerning the household’s ownership of a number of consumer items such as a television and car; dwelling characteristics such as flooring material; type of drinking water source; toilet facilities; and other characteristics that are related to wealth status. To calculate the wealth index, each household asset or characteristic is assigned a weight or factor score determined through principal components analysis (i.e. a statistical tool that condenses a series of variables into one simple variable). These asset scores are then standardized to follow a standard normal distribution, with a mean of zero and a standard deviation of one. These standardized scores are subsequently utilized to establish the thresholds that define the wealth index categories as poorest, poorer, middle, richer, and richest. Further details on generating the wealth index variable are available at the link: https://dhsprogram.com/topics/wealth-index/index.cfm.

### Covariates

To effectively evaluate the connection between wealth index and the outcome, six relevant socio-demographic factors were identified as covariates and adjusted for in the final logistic model. These variables include age (30–34, 35–39, 40–44, 45–49), residence (rural and urban), level of education (no education, basic, secondary and above), wealth index (poorest, poorer, middle, richer, richest), marital status (never married, married, formerly married), occupational status (unemployed, and employed) type of cooking fuel (clean fuel (liquefied petroleum gas, biogas, solar power, electricity, natural gas), unclean fuel (charcoal, wood agricultural grass etc.,)).

### Data analysis

Statistical analysis was performed using Stata version 14 to examine the prevalence of prehypertension among women aged 30–49 years in Benin. The characteristics of the respondents were described based on prehypertension status, and a chi-square test was employed to determine statistically significant differences across the categories of the explanatory variables. Further, we employed binary logistic regression models (i.e., both bivariate and multivariate logistic regression models) to assess the adjusted risk factors associated with the study outcomes. We calculated both crude and adjusted odds ratios (OR) along with 95% confidence intervals (CI). To address any potential sampling bias stemming from under or over-sampling of respondents within the overall population, we applied individual weight variables (v005) from the dataset to all descriptive estimates. Prior to the multivariate regression, we evaluated the possibility of multicollinearity by examining the variance inflation factor (VIF) [20, 21]. The mean VIF score of 6.48 indicated the absence of significant multicollinearity in our analysis.

## Results

### Distribution of prehypertension status by wealth index and other background characteristics

Table 1 displays the proportional distribution of prehypertension status by wealth index and other background characteristics. Overall, out of the 2,787 respondents, approximately one-fifth (~ 19%) were pre-hypertensive. Regarding wealth index, the majority of women in households with the highest wealth index have prehypertension (24.5%) than those in households with the poorest wealth index (17.1%). Similarly, a larger proportion of women in the later age of reproduction (45–49 years) have prehypertension compared to those aged 30–34 years. Concerning the type of cooking fuel, a higher proportion of women who were using clean fuel were pre-hypertensive (~ 32%) than those who were using unclean fuel (18.1%). Wealth status, age, type of residence and type of cooking fuel were the only variables that showed significant differences in their chi-square test results.

**Table 1:** Proportional distribution of prehypertension status by wealth index and other background characteristics

Explanatory variables	Pre-hypertension status	
	Normotension n (%)	Prehypertension n (%)
Overall	2,209(71.75)	578(18.78)
**Wealth index**	X^2^ = 31.3442, P < 0.001	
Poorest	436(72.90)	102(17.09)
Poorer	465(74.38)	93(14.92)
Middle	414(74.29)	91(16.28)
Richer	453(72.36)	127(20.28)
Richest	441(65.63)	165(24.55)
**Covariates**		
**Age**	X^2^ = 26.4672, P < 0.001	
30–34	761(76.62)	162(16.36)
35–39	653(71.79)	169(18.57)
40–44	406(68.32)	125(21.01)
45–49	388(66.88)	122(20.97)
**Educational level**	X^2^ = 6.1152, P = 0.410	
No education	1,637(72.21)	414(18.25)
Basic education	349(71.19)	96(19.56)
Secondary	197(70.92)	56(20.09)
Higher	25(59.30)	13(29.71)
**Occupation status**	X^2^ = 2.5275, P = 0.283	
Unemployed	242(74.56)	51(15.87)
Employed	1,967(71.42)	527(15.87)
**Type of residence**	X^2^ = 19.1465, P < 0.001	
Urban	913(68.54)	298(22.37)
Rural	1,296(74.20)	280(16.05)
**Marital status**	X^2^ = 9.1668, P = 0.057	
Never married	28(64.21)	11(26.35)
Married	1,984(72.04)	500(18.14)
Formerly married	196(70.15)	67(23.92)
**Type of cooking fuel**	X^2^ = 16.7707, P < 0.001	
Clean fuel	78(56.06)	45(31.99)
Unclean fuel	2,130(72.50)	533(18.15)

Note: estimates are weighted

Bivariate and multivariate logistic regression model of prehypertension status among women aged 30–49 years in Benin.

Table 2 shows the association between wealth status and prehypertension. The findings revealed a significant positive association between wealth index and the risks of prehypertension. Women in the richest wealth index were more likely [AOR = 1.4; 95%CI: 1.26,2.26] to have prehypertension compared to their counterparts in the poorest wealth index. Regarding age, women in their later age of reproduction (45–49 years) were more likely to be pre-hypertensive [AOR = 1.5; 95%CI: 1.15–1.98] compared to the reference group (30–34 years). Surprisingly, women who used unclean cooking fuel were less likely to be pre-hypertensive compared to those using clean cooking fuel [AOR = 0.6; 95%CI: 0.37,0.87].

#### Model fitness

In both Model 1 and Model 2, the p-values of the probability chi-square are less than 0.001, indicating that the models are statistically significant. This suggests that at least one variable in each model is associated with the prehypertension status among women aged 30–49 years. In Model 1, the Pseudo R is 0.0065, and in Model 2, it increases to 0.0168. While these values are relatively low, an increase from Model 1 to Model 2 suggests that the additional covariates in Model 2 contribute to a slightly better fit. However, the overall explanatory power of the models appears limited. The AIC decreases from 4795.337 in Model 1 to 4789.52 in Model 2. This suggests that the addition of covariates in Model 2 improves the model’s fit, as a lower AIC is generally preferred.

**Table 2:** Bivariate and multivariate logistic regression model of prehypertension status among women aged 30–49 years in Benin

Variables	Model 1	Model 2
	Crude odds ratio (COR)	Adjusted odds ratio (AOR)
Wealth index		
Poorest	Ref	Ref
Poorer	0.9[0.67,1.23]	0.9[0.75,1.32]
Middle	1.0[0.72,1.34]	1.0[0.69,1.24]
Richer	1.25[0.93,1.67]	1.2[0.97,1.67]
Richest	1.67***[1.27,2.20]	1.4*[1.26,2.26]
**Covariates**		
**Age**		
30–34		Ref
35–39		1.2[0.95,1.55]
40–44		1.4**[1.10,1.87]
45–49		1.5**[1.15,1.98]
**Educational level**		
No education		Ref
Basic education		0.9[0.71,1.21]
Secondary		0.7[0.51,1.04]
Higher		0.9[0.47,1.94]
**Occupational status**		
Unemployed		Ref
Employed		1.3[0.91,1.94]
**Type of residence**		
Urban		Ref
Rural		0.8[0.66,1.01]
**Marital status**		
Never married		Ref
Married		0.7[0.36,1.48]
Formerly married		0.9[0.44,2.02]
**Type of cooking fuel**		
Clean fuel		Ref
Unclean fuel		0.6*[0.37,0.87]
***Model fitness***		
Prob > chi2	< 0.001	< 0.001
Pseudo R	0.0065	0.0168
AIC	4795.337	4789.52

AIC: Akaike Information criterion

## Discussion

In this study, we investigated the association between wealth index and the risk of prehypertension among women aged 30–49 years in Benin. Consistent with previous studies conducted in South Asia [14] and Bangladesh [22], our study found a positive association between wealth index and prehypertension risk. That is, women in the richest wealth index were 1.4 times more likely to have prehypertension compared to those in the poorest wealth index. This finding is inconsistent with Chambergo-Michilot et al.’s [15] that found the risk of prehypertension to significantly reduce as wealth increases. Our finding is inconsistent with a study conducted in Algeria [11] that found no significant association between wealth index and prehypertension risk. We postulate that the observed association between wealth status and prehypertension may be due to the point that affluent women may be more inclined to adopt sedentary lifestyles [22]. The findings underscore a need for the Ministry of Health in Benin to prioritize women in affluent communities and households in their health education and prehypertension awareness campaigns.

Consistent with previous studies conducted in China [1], Algeria [11], Bangladesh [22], and Peru [23], our study revealed that the risk of prehypertension increases with age. This is corroborated by the findings of a systematic review that investigated the determinants of prehypertension in Africa [9]. Biologically, ageing exacerbates impaired arterial functions which increases blood pressure [23]. Given that the highest risk of prehypertension was found among women aged 45–49 years, it is possible that this risk may be due to estrogen deficiencies that occur during the menopausal transition (usually beginning at age 45) [24]. Hence, resulting in vascular dysfunction and increasing women’s chances of developing prehypertension [25].

We found a significant association between type of cooking fuel used and the risk of prehypertension – a result that is corroborated by Dutta et al. [26]. However, this association deviated from what was expected. That is, contrary to previous studies that show that the use of unclean cooking fuel exacerbates the risk of cardiovascular diseases including hypertension and prehypertension [27, 28], we found the reverse where unclean cooking fuel use was associated with a lower risk of prehypertension. It is uncertain the reasons for the observed association, and thus, calls for more comprehensive studies such as randomized controlled studies to fully understand the extent and pathways through which type of cooking fuel contributes to prehypertension risk.

### Strengths and limitations

This study was based on a large, nationally representative dataset. For that matter, the findings are generalizable to the wider population of middle-aged women of reproductive age in Benin. Additionally, the study used appropriate statistical analysis; thus, guaranteeing the authenticity and validity of our results. As a study based on secondary data, we were limited in the kind of covariates that could be controlled for in the multivariate logistic regression model. For example, the data does not have any variable that measures the physical activeness, abdominal obesity, hyperglycemia, and sedentary lifestyle of the respondents. Also, the design of the demographic and health survey precludes the establishment of causal inferences between wealth status, other significant covariates and prehypertension risk. Although body mass index was present in the dataset to measure obesity, it was intentionally excluded due to the high number of missing variables which has the potential to significantly underestimate the phenomenon.

## Conclusion

Based on the findings, we conclude that wealth status is a significant predictor of prehypertension among women in Benin. Therefore, the Ministry of Health in Benin should prioritize health education and prehypertension awareness campaigns specifically targeting women in affluent communities and households. These campaigns should focus on promoting healthy dietary choices and encouraging physical activity to mitigate the elevated risk associated with wealth status. Recognizing the influence of age on prehypertension risk, it is imperative for older women of reproductive age to be targeted as primary beneficiaries of health education programs and prehypertension screening programs. Given that unclean cooking fuel use was associated with lower prehypertension risk in this study, policymakers should prioritize conducting more comprehensive research, including randomized controlled studies. These studies should aim to understand the mechanisms and pathways through which cooking fuel type influences prehypertension risk.

## Data Availability

The datasets generated and/or analysed during the current study are available in the Measure DHS repository: http://dhsprogram.com/data/available-datasets.cfm.

## Abbreviations

AOR: Adjusted Odds Ratio
BDHS: Benin Demographic and Health Survey
CI: Confidence Interval
WHO: World Health Organization
